# Adrenergic Stress Protection of Human iPS Cell-Derived Cardiomyocytes by Fast K_v_7.1 Recycling

**DOI:** 10.3389/fphys.2017.00705

**Published:** 2017-09-14

**Authors:** Ilaria Piccini, Edda Fehrmann, Stefan Frank, Frank U. Müller, Boris Greber, Guiscard Seebohm

**Affiliations:** ^1^Department of Cardiovascular Medicine, Institute of Genetics of Heart Diseases, University of Münster Medical School Münster, Germany; ^2^Human Stem Cell Pluripotency Laboratory, Max Planck Institute for Molecular Biomedicine Münster, Germany; ^3^Institute of Pharmacology and Toxicology, University of Münster Münster, Germany; ^4^Chemical Genomics Centre of the Max Planck Society Dortmund, Germany

**Keywords:** adrenergic stress, KCNQ1, ion channel transport, fast recycling, stress-induced arrhythmia, hiPSC-derived cardiomyocytes

## Abstract

The fight-or-flight response (FFR), a physiological acute stress reaction, involves positive chronotropic and inotropic effects on heart muscle cells mediated through β-adrenoceptor activation. Increased systolic calcium is required to enable stronger heart contractions whereas elevated potassium currents are to limit the duration of the action potentials and prevent arrhythmia. The latter effect is accomplished by an increased functional activity of the K_v_7.1 channel encoded by *KCNQ1*. Current knowledge, however, does not sufficiently explain the full extent of rapid K_v_7.1 activation and may hence be incomplete. Using inducible genetic *KCNQ1* complementation in *KCNQ1*-deficient human induced pluripotent stem cell-derived cardiomyocytes (hiPSC-CMs), we here reinvestigate the functional role of K_v_7.1 in adapting human CMs to adrenergic stress. Under baseline conditions, K_v_7.1 was barely detectable at the plasma membrane of hiPSC-CMs, yet it fully protected these from adrenergic stress-induced beat-to-beat variability of repolarization and *torsade des pointes*-like arrhythmia. Furthermore, isoprenaline treatment increased field potential durations specifically in KCNQ1-deficient CMs to cause these adverse macroscopic effects. Mechanistically, we find that the protective action by K_v_7.1 resides in a rapid translocation of channel proteins from intracellular stores to the plasma membrane, induced by adrenergic signaling. Gene silencing experiments targeting RAB GTPases, mediators of intracellular vesicle trafficking, showed that fast K_v_7.1 recycling under acute stress conditions is RAB4A-dependent.Our data reveal a key mechanism underlying the rapid adaptation of human cardiomyocytes to adrenergic stress. These findings moreover aid to the understanding of disease pathology in long QT syndrome and bear important implications for safety pharmacological screening.

## Introduction

Emotional and physical stress reactions involve hypothalamic structures leading to an activation of the sympathetic nervous system (SNS). Moreover, several direct and feedback interactions between the hypothalamus, the pituitary gland, and the adrenal (suprarenal) glands constitute the hypothalamic-pituitary-adrenal axis (HPA axis). The HPA axis controls stress reactions and regulates many body processes including cardiac function. The mediator hormone released from the adrenal gland is cortisol. Cortisol stimulates several proteins including SGK1 which, besides many other effects, is thought to control cardiac tissue homeostasis by increasing plasma membrane densities of key ion channels—most notably of the composite delayed rectifier channel K_v_7.1/KCNE1, a key repolarizing component of cardiac action potentials (Seebohm et al., 2007).

Whereas the HPA axis is relevant for homeostasis, acute stress regulation in course of the so-called fight-or-flight response (FFR) is achieved via the SNS. SNS regulation of cardiac function is mediated by (nor)adrenaline that primarily activates β-receptors. Activation of β1-receptors during FFR increases the heart rate (chronotropy) through pacemaker cells and stimulates force development (inotropy) (Bers, 2002). Both effects require a physiological adaptation of the working myocardium supposedly again through a modulation of K_v_7.1/KCNE1 function, which would have to occur very rapidly during FFR initiation. Mechanisms underlying the putative short-term regulation of K_v_7.1/KCNE1 are essentially unexplored in human cardiomyocytes, though, largely because these cells are barely accessible for experimental purposes. In addition, there are species differences regarding K_v_7.1/KCNE1 physiology (Nerbonne, 2000). Based on *in-vitro* studies, it has been shown in this context that β-adrenergic stimulation impacts on K_v_7.1/KCNE1 channel kinetics (Marx et al., 2002). These positive effects, however, are not extensive enough to explain the full extent of K_v_7.1/KCNE1 stimulation by β-adrenergic activation (Nicolas et al., 2008).

Conversely, an impairment of K_v_7.1/KCNE1 function due to genetic mutations leads to increased ventricular action potential durations in patients, which becomes apparent as a prolongation of the QT interval in the electrocardiogram. Loss-of-function mutations in the K_v_7.1-encoding gene *KCNQ1* account for ~40% of long QT syndromes with known genetic basis (LQT1) (Splawski et al., 2000). LQT1 predisposes for torsade de pointes arrhythmias and sudden cardiac death. The typical trigger of these severe arrhythmias in LQT1 is physical or emotional stress, highlighting that K_v_7.1 function is particularly critical under β-adrenergic stimulation conditions—and might itself be subject to modulation by this pathway via a yet-to-be discovered mechanism. hiPSCs can be converted into functional - albeit relatively immature—CMs (Burridge et al., 2012). Hence, hiPSC-derived CMs may serve as a powerful system for studying such fundamental questions of cardiac ion channel physiology, and that of K_v_7.1 in particular. Indeed, the physiological relevance of this system is underscored by patient-specific disease models proven to recapitulate pathological phenotypes of LQT1 as well as of its most severe variant, Jervell and Lange-Nielsen syndrome (JLNS) (Moretti et al., 2010; Zhang et al., 2014).

A coordinated cellular adaptation to an external stimulus like adrenergic stress depends not only on the presence of the mediator protein within the cell but also on its appropriate subcellular localization, like the actual presence of K_v_7.1 at the CM plasma membrane. In living cells, intracellular vesicles can serve as reservoirs of membrane proteins including ion channels to harbor these in situations when not required at the cell surface. These vesicles are called early endosomes. Another class of vesicles termed recycling endosomes are located in proximity to the plasma membrane allowing for fast fusion with selfsame to increase protein abundance at the cell surface. Small GTPases of the RAB protein family, the ubiquitin ligase NEDD4-2, as well as intracellular Ca^2+^ elevation have been implicated in triggering K_v_7.1/KCNE1 trafficking events in heterologous expression systems and animal models (Seebohm et al., 2007; Wang et al., 2013; Andersen et al., 2015). It is unclear, though, if these mechanisms represent the physiological reality in human cardiomyocytes, particularly under acute stress conditions.

Here, we employ a genetic system complementing K_v_7.1-deficiency in a hiPSC model of JLNS. We utilize these cells to reinvestigate the role of K_v_7.1 in adapting human cardiomyocytes to adrenergic stress and uncover a novel key regulatory mechanism underlying K_v_7.1/KCNE1 activation via the β-adrenergic system.

## Materials and methods

### Genetic manipulation of hiPSCs and hiPSC-cardiomyocytes

**Figure 1:**
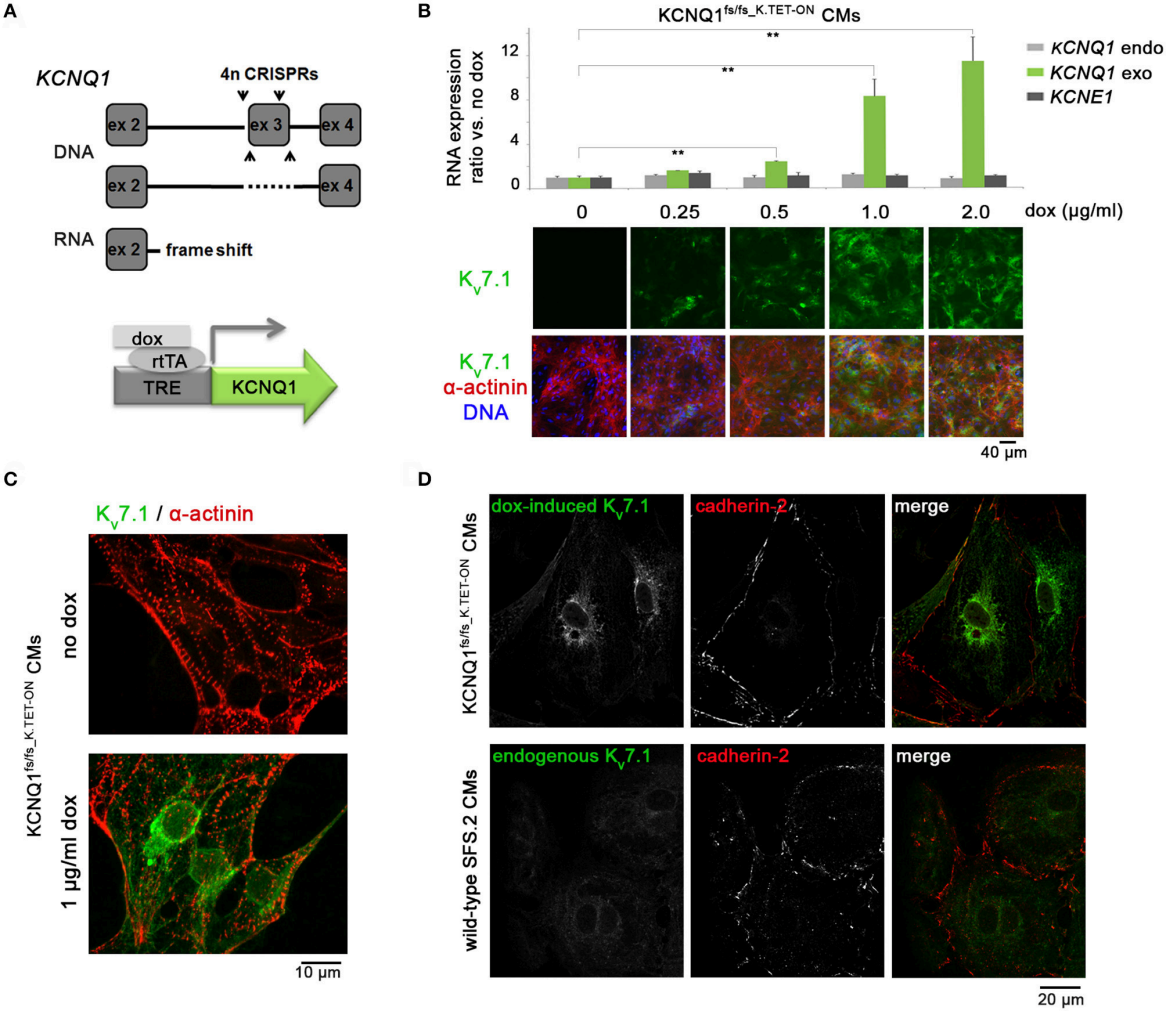
Generation and validation of KCNQ1^fs/fs_K.TET−ON^ hiPSC-CMs. **(A)** Top: Illustration of genomic *KCNQ1* disruption strategy using CRISPR vectors deleting exon 3 to cause a homozygous frame shift and premature stop at the RNA level. Bottom: Schematic of genetic rescue by PiggyBac transposition-mediated integration of a doxycycline-inducible *KCNQ1* transgene. **(B)** Top: Dox titration in KCNQ1^fs/fs_K.TET−ON^ hiPSC-derived CMs showing dose-dependent *KCNQ1* induction at RNA level (*n* = 3 biological replicates, ^**^p < 0.01). Bottom: Confirmation at protein level also highlighting overall high CM differentiation efficiencies as evidenced by α-actinin staining. 1 μg/ml dox was used in subsequent experiments throughout. **(C)** K_v_7.1 staining in α-actinin-positive cardiomyocytes showing homogeneous induction by dox and complete absence of the channel in untreated CMs. **(D)** Top: Under baseline conditions, dox-induced K_v_7.1 on KCNQ1^fs/fs^ background shows a predominantly perinuclear staining pattern with little abundance at the outer CM membrane marked by cadherin-2. Bottom: Endogenous K_v_7.1 in 5 week-old wild-type SFS.2 CMs shows, despite faint staining, a similar subcellular localization.

For disrupting *KCNQ1* in wild-type SFS.2 hiPSCs, a validated integration-free line derived from foreskin fibroblasts (Zhang et al., 2014), (“wt1” therein) 4 guide RNAs were designed to target sequences around the intron 2/exon 3 and exon 3/intron 3 splice junctions as illustrated in (Figure 1A). The corresponding DNA sequences are given in Table S1. These were cloned as double-stranded oligonucleotides into a CRISPR/Cas9n nickase vector containing a GFP-2A-puromycin selection cassette as described (modified Addgene plasmid # 42335) (Cong et al., 2013; Zhang et al., 2014). All 4 CRISPR vectors were transfected into SFS.2 hiPSCs using Fugene HD (Roche) and transiently selected for 24 h using 0.5 μg/ml puromycin to enrich for transfected cells. Following replating of the cells at clonal dilution, clonal half-colonies were picked another ~2 weeks later, to be PCR-screened for successful *KCNQ1* exon 3 excision which additionally causes a premature frame-shift.

A sequence-validated *KCNQ1* knockout hiPSC clone was expanded, verified to show sustained cardiomyocyte differentiation competence, and complemented thereafter by an inducible *KCNQ1* transgene. Briefly, the corresponding reading frame was subcloned from a pre-existing *KCNQ1-GFP*-containing vector into the *Spe I* and *Nhe I* sites of a modified KA0717 PiggyBac TET-ON vector (originally a gift from Dr. Kenjiro Adachi). This construct was then cotransfected together with transactivator and transposase-encoding vectors (KA0637 and SBI Biosciences # PB200PA-1) into KCNQ1^fs/fs^ hiPSCs using Fugene HD at a DNA mass ratio of 15:1:3, respectively. Stable transgene-positive clones were selected for 1 week using 50 μg/ml G418. One selected clone was expanded, verified to homogeneously up-regulate *KCNQ1* upon doxycycline administration (1 μg/ml), and validated to show sustained cardiomyocyte differentiation competence following these manipulation steps. In dox-treated samples throughout this study, KCNQ1 was induced for at least 2 days prior to performing experiments, which ensured equilibrated transgene levels in the cells. Dox-untreated cells of the same cell line served as negative controls throughout.

For overexpression using self-replicating *in-vitro* transcribed RNA transgenes, *DsRed1-hRAB* fusion ORFs were gene-synthesized and cloned into a T7-VEE vector (Table S1; Addgene plasmid # 58970) (Yoshioka et al., 2013), upstream of an IRES-puromycin cassette. Messenger RNA was generated as previously described (Yoshioka et al., 2013), by applying an Ambion Megascript T7 *in vitro* transcription kit to the linearized DNA constructs, as well as Cellscript kits # C-SCCE0610, # C-SCMT0610, and # CPAP5104H for RNA capping, 2′-O-Methylation, and PolyA tailing, respectively. Purified polycistronic RNA was transfected into freshly replated cardiomyocytes using Ribojuice (Merck; 250 ng RNA: 1 μl Ribojuice: 1 μl Boost reagent in 50 μl OptiMEM per 200,000 cells in 24-well format). Addition of 100 μg/ml B18R (Bioscience) to the cultures served to sustain cell viability during and after transfection which was carried out in the absence of antibiotics. 0.2 μg/ml puromycin was transiently applied from 1 day post transfection to enrich for transgene-positive cells.

For siRNA-mediated knockdowns, Dharmacon ON-TARGETplus SMARTpools (# L008539000005, # L004009000005, # L010388000005, L004726000005, and non-targeting control pool # D-001810-10-05) were transfected into freshly replated hiPSC-CMs at 25 pmol: 4 μl Ribojuice siRNA transfection reagent (Merck, in 100 μl OptiMEM) per 500,000 cells in 12-well format. As above, B18R supplementation to the culture medium served to sustain cell viability during and after transfection which was carried out in the absence of antibiotics. The success of RAB silencing was validated 24 h after transfection using *RAB* qPCR primers given in Table S1. Immunostaining analysis following short-term pharmacological treatment with adrenergic/cAMP stress inducers was performed 48 h after transfection.

### hiPSC culture

SFS.2 hiPSCs and the KCNQ1^fs/fs_K.TET−ON^ derivative line were routinely cultured in six-well plates on 1:75 diluted Matrigel™ HC (Corning # 354263), in defined FTDAC medium (Frank et al., 2012). FTDAC consisted of DMEM/F12, 1 × ITS (Corning # 354350), 0.1% human serum albumin (Biological Industries # 05-720-1B), 1 x defined lipids (Thermo), 1 × penicillin/streptomycin/L-glutamine (PSG), 10 ng/ml FGF2 (PeproTech # 100-18B), 0.2 ng/ml TGFβ1 (eBioscience # 34-8348-82), 50 nM Dorsomorphin (Santa Cruz # sc-200689), 5 ng/ml Activin A (eBioscience # 34-8993-85), and 1 nM C-59 (Tocris # 5148/10). Cells were routinely passaged at 700,000 single cells per six-well following Accutase™ digestion with 10 μM Y-27632 (abcamBiochemicals # ab120129). This way, hiPSCs were kept in culture for a maximum of 30 passages shown to preserve an intact karyotype (Zhang et al., 2015).

### Cardiac differentiation and maintenance of cardiomyocytes

Cardiac induction was performed under serum and serum albumin-free conditions as optimized previously (Zhang et al., 2015): Fully confluent hiPSC cultures were harvested using Accutase, resuspended in day 0 differentiation medium, and seeded out at 700,000 cells per well of a Matrigel-coated 24-well plate, in a total volume of 2 ml. Day 0 differentiation medium consisted of KO-DMEM, 1 × ITS, 10 μM Y-27632, PSG, 5 ng/ml Activin A, 10 ng/ml FGF2, 0.5–1 ng/ml BMP4 (R&D # 314-BP-050), and 1 μM CHIR99021 (AxonMedchem # Axon 1386). Medium in differentiation plates was exchanged on a daily basis. From day 1 onwards, the basal differentiation medium consisted of KO-DMEM, 1 × TS (transferrin/selenium), 250 μM 2-phospho-ascorbate (Sigma-Aldrich # 49752), and PSG. 100 × TS stock was prepared in advance by dissolving 55 mg transferrin (Sigma # T8158) in 100 ml PBS containing 0.067 mg sodium selenite (Sigma # S5261). 0.2 μM WNT inhibitor C-59 was added to the cultures from 48 to 96 h of differentiation to promote cardiac specification.

Beating monolayers emerging from day 6 of differentiation were dissociated using TrypLE Select (Thermo) and replated between days 8–10 at a ratio of ~1:4 using CM splitting medium consisting of RPMI 1640 (Thermo), 1 × ITS, 0.2% HSA, 250 μM phospho-ascorbate, 0.008% thioglycerol, PSG, and 10 μM Y-27632. Next day, medium was replaced by CM maintenance medium consisting of KO-DMEM, 1 × ITS, 0.2% HSA, 250 μM phospho-ascorbate, 0.008% thioglycerol, and PSG. CMs maintained for culture-induced maturation under these conditions for at least 1 week were then re-replated for experiments on plastic surfaces, glass cover slips, or multielectrode arrays (all coated with diluted Matrigel), as appropriate. Responses to isoprenaline required hiPSC-CM differentiation/maturation for a total of ~3.5 weeks, whereas forskolin treatments could be performed at an earlier stage (~2.5 weeks).

### Electrophysiological analysis on MEAs

For electrophysiological analyses on microelectrode arrays (USB-MEA256 system, Multichannel Systems), the electrode areas of plasma-cleaned 9-well MEAs were coated with 3 μl of a 1:150 diluted Matrigel/0.1% gelatin solution in KO-DMEM for ~2 h at 37°C in a humidified cell culture incubator. hiPSC-CMs were dissociated from maintenance cultures using 10 x TrypLE Select. Coating solution was removed from the electrode arrays to be replaced by 25,000–50,000 cells resuspended in a ~3 μl droplet of CM replating medium. CMs were allowed to attach for ~30 min. Subsequently, MEA chambers with attached cells were filled with 150 μl of CM replating medium. From the following day onwards, cell preparations were used for electrophysiological recordings at 37°C. Recordings of drug-treated cells were initiated after a brief wash-in time (isoprenaline, fresh: 1 μM, forskolin: 10 μM, IBMX: 100 μM, propranolol: 10 μM, prazosin: 10 μM). Algorithms implemented in MC Rack software v4.5.7 allowed for determining field potential durations (FPDs/QT_max_ intervals) and peak-to-peak (RR) intervals. If appropriate, data were merged from consecutive measurements of a given sample and averaged between independent replicates. Data were processed in MS Excel where QT_max_-like intervals were frequency-corrected using Bazett's formula: QTc_max_ = QT_max_ [ms]/(RR [s])^0.5^. *Poincaré* plots representing the beat-to-beat variability of repolarization were generated from consecutive beats of representative recordings. Short-term varitation was defined as the sum of all ΔQT_max_ differences from 30 consecutive beats in a representative sample, divided by 30^0.5^.

### Calcium imaging

hiPSC-CMs were replated onto Matrigel-coated glass slides and maintained in CM maintenance medium. At 3 weeks, the cells were transferred into a perfusion-stimulation-chamber and incubated with 9 μM Indo-1 acetoxymethyl ester (Thermo) for 15 min at room temperature. After mounting the perfusion-stimulation-chamber on an inverted Nikon Ti-S fluorescence microscope, the cells were washed with Tyrode's solution (140 mM NaCl, 5.8 mM KCl, 0.5 mM KH_2_PO_4_, 0.4 mM Na_2_HPO_4_, 0,9 mM MgSO_4_, 10 mM HEPES, 11.1 mM Glucose, 2 mM CaCl_2_, pH 7.3) in order to minimize background fluorescence. Next, intracellular Ca^2+^ transients were evoked via field-stimulation at a frequency of 0.5 Hz. The Ca^2+^ transients were recorded as the Indo-1 fluorescence emission ratio F405 nm/F495 nm using an IonOptix (Milton, MA) detection system (excitation: 344 nm). At least 10 cell clusters per glass slide were measured under basal conditions as well as during superfusion with isoprenaline (1 μM). For each cell cluster, ten intracellular Ca^2+^ transients were averaged using the IonWizard analysis program to determine changes in peak amplitude and transient decay kinetics.

### Immunoblotting, immunocytochemistry, and image analysis

Immunoblotting was performed according to standard procedures. Immunofluorescence analysis was carried out on glass cover slips following formaldehyde or aceton-ethanol fixation, and permeabilization / blocking with 0.2% Triton X-100/5% FCS/2% BSA/2% glycine in PBS for 45 min. Primary antibodies incubated over night were: α-actinin (Sigma # A7811, 1:800), DsRed (Chromotek # 5f8-100, 1:500, for DsRed-RAB fusion transgenes), K_v_7.1 (WB: Alomone Labs # APC-022, 1:250; IF: Santa Cruz # sc-20816, 1:100), cadherin-2 (BD # 610920, 1:200), and NKX2.5 (R&D # AF2444, 1:1,000). Secondary antibodies were appropriate Alexa-488 or 568-conjugated ones (Thermo). Images shown are full or cropped frames taken with a Zeiss Axio Imager or a Leica confocal microscope with 63 x lens.

Stacked confocal images were analyzed using Leica LAS AF Lite software. Focusing on areas with clear cadherin-2 staining, 3 μm long regions of interest (ROIs) crossing the outer membrane in a perpendicular fashion were defined in an unbiased manner and in arbitrary orientations. These ROIs randomly covered outer as well as inner cells of CM clusters. Absolute red and green fluorescence intensities were extracted at an eight-bit scale and normalized by dividing these values by 256. In MS Excel, the maximum red (cadherin-2) values in each series were defined as the plasma membrane centers. Values extending beyond 1.25 μm left and right to these points were discarded. 10–30 analyzed ROIs were merged for each given image/sample, which was defined as one biological replicate. Mean values and statistics were based on 4–8 such independent replicates per sample type.

### RT-qPCR

RNA was isolated using NucleoSpin RNA kits with on-column DNA digestion (Machery Nagel). Reverse transcription was performed using M-MLV reverse transcriptase (Affymetrix # 78306) with oligo-dT_15_ priming at 42°C. Real-time PCR was carried out using efficiency-validated primers given in Table S1 and BioRad iTaq™ Universal SYBR Green Supermix (# 172-5124) on an ABI 7500 cycler. *RPL37A* or *CTNT* served as housekeeping controls. Data are expressed relative to an indicated control sample.

### Statistical analysis

Unless stated otherwise, data are presented as means between biological replicates ± SEM. Pairs of treated samples and controls were compared using two-sided unpaired *t*-tests, or Mann-Whitney rank sum tests in case of calcium imaging analysis. In case of the dox titration experiments, one-way ANOVA served to additionally confirm the overall dox dependency of the *KCNQ1* transgene. For image analysis following confocal immunocytochemistry, 10–30 cross-membrane regions were analyzed per image and merged based on extracted intensities. 4–8 images from 2 to 3 independent experiments then formed the basis for determining mean normalized pixel intensities and statistics. A *p*-value of < 0.05 (^*^) or < 0.01 (^**^) was considered statistically significant as indicated.

## Results

### Generation of a KCNQ1-deficient hiPSC model with inducible transgenic complementability

To explore the stress-protective role of K_v_7.1 in a human cardiac cell context (Zhang et al., 2014), we sought to generate a transgenic TET-ON model on *KCNQ1*-deficient hiPSC background. This approach would bear the advantages of detecting K_v_7.1 in a technically less demanding manner while allowing for direct comparisons between K_v_7.1-positive and deficient CMs in otherwise identical cell preparations. Moreover, a transgenic approach could avoid otherwise lengthy and impractical maturation periods required for visualizing endogenous K_v_7.1 (Zhang et al., 2014, 2015). Hence, targeted genomic deletions causing a frameshift and premature stop codon at the RNA level were induced on both *KCNQ1* alleles of integration-free hiPSCs using CRISPR/Cas9n (Cong et al., 2013). Subsequently, PiggyBac transposition was employed to stably integrate an inducible *KCNQ1* transgene in these cells (Figure 1A). A clonal cell line termed KCNQ1^fs/fs_K.TET−ON^ was validated to differentiate well into cardiomyocytes employing an established differentiation protocol (Zhang et al., 2015) and to express *KCNQ1* in a doxycycline (dox) concentration-dependent manner (Figure 1B). A minimal dox dose (1 μg/ml) leading to KCNQ1 expression in most CMs was employed in all subsequent experiments (Figure 1C).

Although it is beyond doubt that K_v_7.1 exerts some basal function in cardiac cells under ground state conditions (Greber et al., 2015), we noticed with interest that very little K_v_7.1 protein was actually localized to the outer cardiomyocyte membrane (Figure 1D, top panels). Indeed, the concept that K_v_7.1 plays a prominent role in unstressed cardiomyocytes, including hiPSC-CMs, is controversial (Christ et al., 2015). However, it cannot be fully ruled out that transgenic K_v_7.1 shows any non-physiological behavior. Hence, as crucial control, we also stained for endogenous K_v_7.1 in wild-type hiPSC-CMs of the otherwise same genetic background, following *in-vitro* maturation over 5 weeks. Despite showing an overall faint staining as expected, it was evident again that K_v_7.1 was mainly localized to the cyotoplasm (Figure 1D, bottom panels). Thus, transgenic K_v_7.1 displayed a very similar staining pattern compared to endogenous K_v_7.1 in maturated wild-type hiPSC-CMs and this is characterized by low outer membrane abundance.

### K_v_7.1 protects hiPSC-CMs from adrenergic stress-induced torsade de pointes

**Figure 2:**
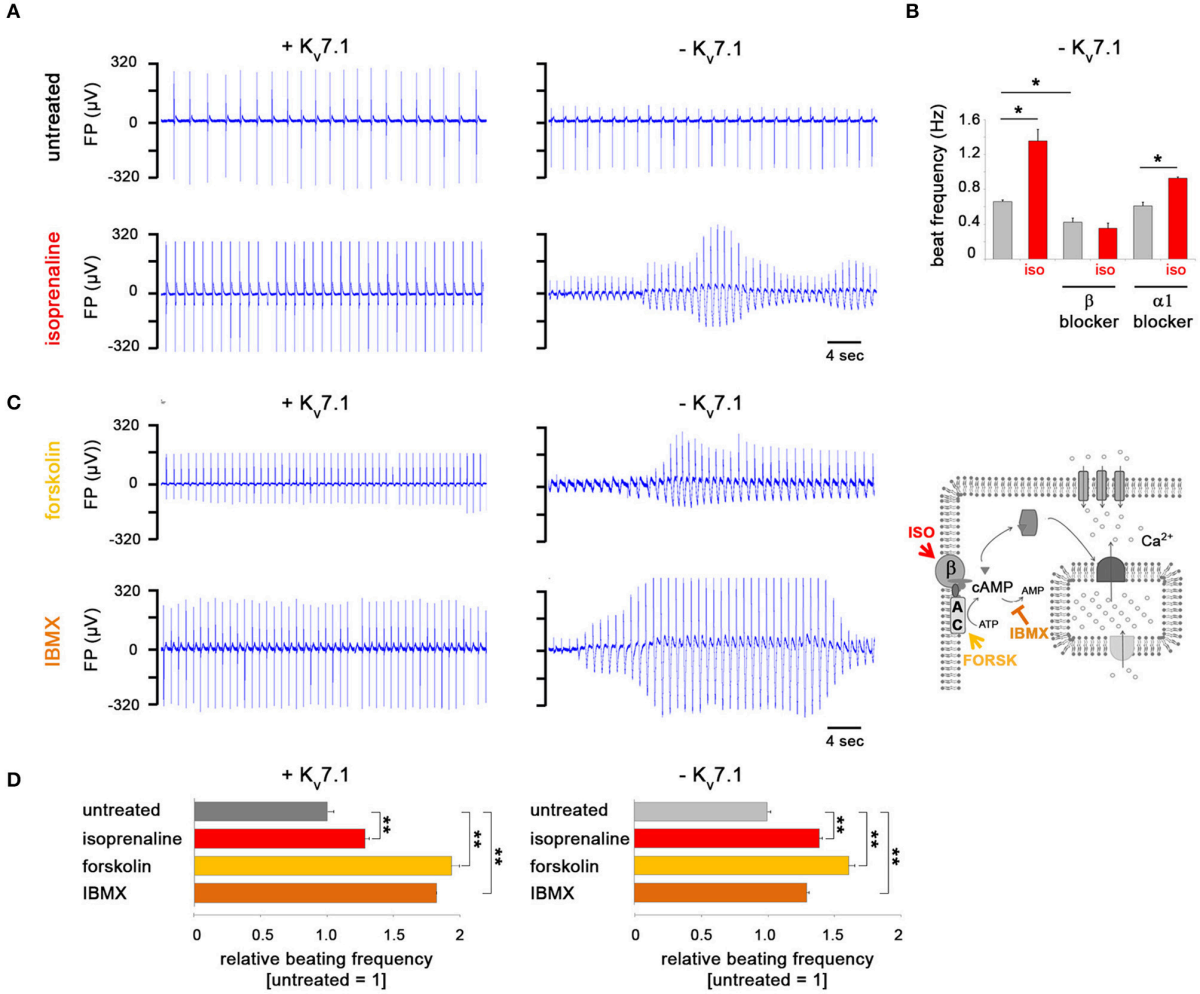
K_v_7.1 protects hiPSC-CMs from *TdP*-like arrhythmia under β-adrenergic stress conditions. **(A)** Representative MEA traces showing isoprenaline-induced arrhythmia in the absence of K_v_7.1 and prevention of beating irregularities in its presence. Note the positive chronotropic effect in both cases. **(B)** Chronotropic response by isoprenaline is abolished following β but not α-blocker pretreatment using propranolol or prazosin, respectively (both at 10 μM, *n* = 4, ^*^p < 0.05). **(C)** In the absence of K_v_7.1, cAMP enhancers forskolin (10 μM) and IBMX (100 μM) promote *TdP*-like arrhythmia similar to isoprenaline (left, representative MEA recordings). The illustration on the right depicts the targets of the three cAMP-enhancing molecules used. β: β-adrenergic receptor, AC: adenylyl cyclase. **(D)** Positive chronotropic effect by isoprenaline, forskolin, and IBMX is independent of *KCNQ1* expression (*n* = 4 independent experiments, 6–19 total measurements, ^**^p < 0.01 vs. untreated).

Following up on our previous efforts related to modeling JLNS (Zhang et al., 2014), we next challenged KCNQ1^fs/fs_K.TET−ON^-CMs with adrenergic agonists. Both in dox-treated, that is *KCNQ1*-expressing, cells and in K_v_7.1-deficient ones, isoprenaline administration caused a significant increase in spontanesous beating frequencies on multielectrode arrays (MEAs), demonstrating that the underlying signaling cascade was operative in this system (Figures 2A,B). Strikingly, in K_v_7.1-deficient CMs, isoprenaline additionally caused severe *torsade de pointes*-like (*TdP*) arrhythmias known to be a key life-threatening feature in LQT1 and JLNS patients. Dox-mediated *KCNQ1* induction, however, protected the hiPSC-CMs fully from showing *TdP*-like behavior (Figure 2A). Isoprenaline is known to activate β-adrenoceptors. To rule out that these effects were additionally mediated by α-adrenergic signaling, isoprenaline was supplemented in the presence of specific β and α-blockers. Indeed, the positive chronotropic effect was prevented by propanolol (vs. β) but not by prazosin (vs. α) pretreatment (Figure 2B). Moreover, more immediate activators of cAMP-mediated signaling, forskolin and IBMX (Figure 2C, right), reproducibly caused beating rate increases and *TdPs* in K_v_7.1-deficient CMs similar to isoprenaline (Figures 2C,D). Hence, stimulation of β-adrenergic/cAMP signaling rapidly induced physiological arrhythmia-like behavior in a JLNS-like scenario and K_v_7.1 complementation rescued this effect—although the channel displayed overall low outer membrane abundance under baseline conditions (Figure 1D).

### β-adrenergic stimulation promotes additional QT prolongation specifically in K_v_7.1-deficient CMs

**Figure 3:**
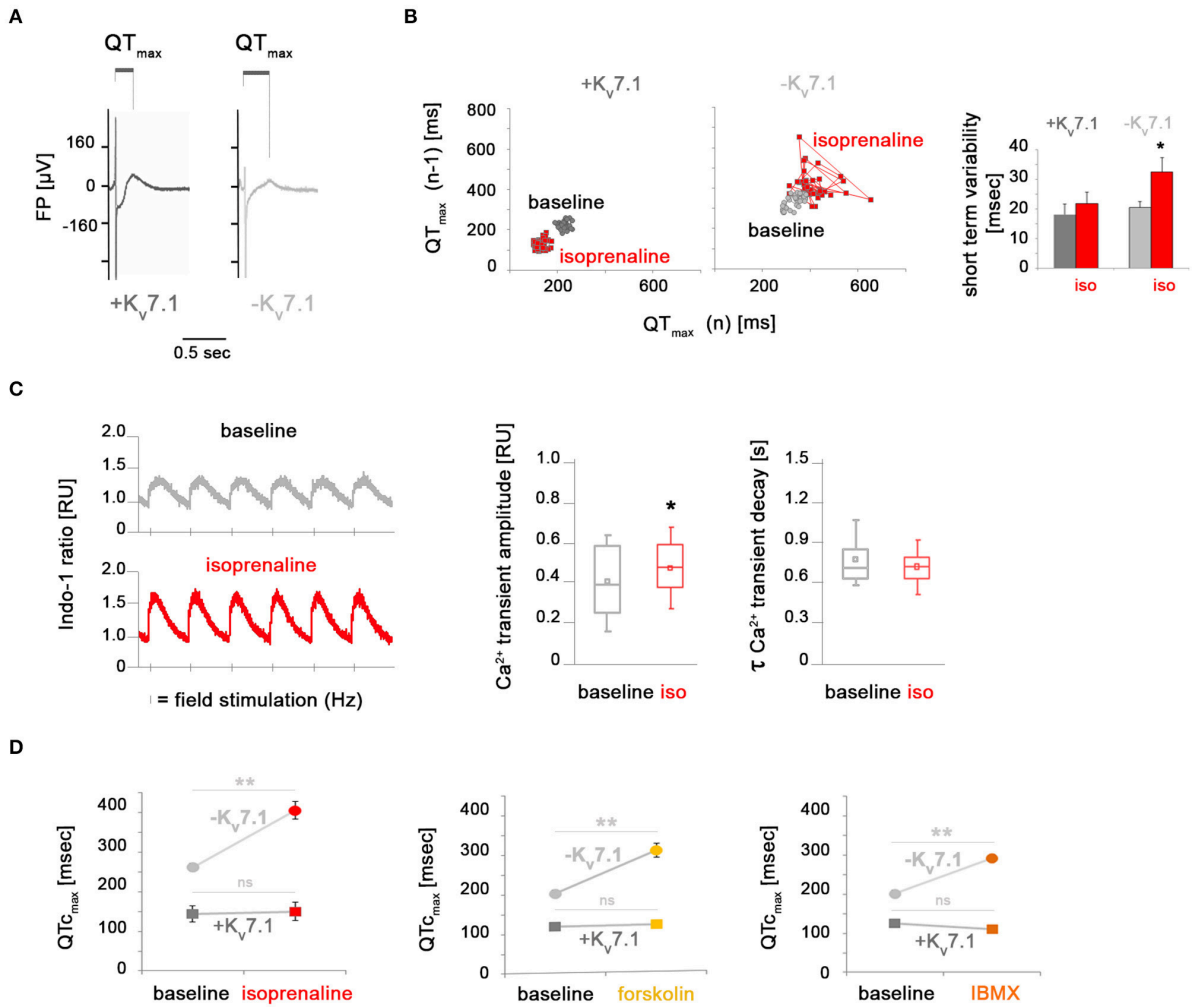
K_v_7.1-dependent and independent effects of β-adrenergic stimulation on beat-to-beat variation, calcium signaling, and field potential duration. **(A)** Representative MEA recordings highlighting QT_max_-like intervals used for quantifying field potential durations. **(B)** Isoprenaline treatment promotes beat-to-beat variability of repolarization in the absence of K_v_7.1 but not in its presence. Left: Representative *Poincaré* plots. Right: Quantification of short-term FPD variation from independent experiments (*n* = 6–15, ^*^p < 0.05 vs. baseline). **(C)** Ca^2+^ transients in KCNQ1^fs/fs^ hiPSC-CMs. Left: Representative recordings of cells stimulated with 0.5 Hz under basal conditions and upon isoproterenol treatment [1 μM]. Right: Quantification of Ca^2+^ transient amplitudes (*n* = 103 cells per condition from four independent preparations each, ^*^p < 0.05). **(D)** Quantification of frequency-corrected FPDs under baseline and adrenergic stress conditions. Activation of cAMP signaling leads to FPD prolongation specifically in K_v_7.1-deficient CMs but not in K_v_7.1-positive ones (four independent experiments, 6–19 total measurements, ^**^p < 0.01 vs. untreated).

To better understand the severe arrhythmia-causing effects by β-adrenergic stimulation in KCNQ1^fs/fs^ CMs, we next monitored the beat-to-beat variability of repolarization (BVR), a key risk parameter for developing *TdPs* (Thomsen et al., 2006; Oosterhoff et al., 2007). This was based on determining field potential durations (QT_max_-like intervals) of consecutive beats on MEAs (Figure 3A). Whereas, *TdP*-like behavior was not seen in every experiment, a pronounced increase in BVR quantified as short-term variability was consistently observed in K_v_7.1-deficient CMs under isoprenaline, but not in KCNQ1-complemented ones (Figure 3B). Increased BVR is related to enhanced Ca^2+^ signaling (Johnson et al., 2013) and therefore, we also performed calcium imaging of K_v_7.1-negative CMs under baseline and stressed conditions. Calcium transient amplitudes were significantly elevated following isoprenaline stimulation, as expected (Figure 3C, left and middle panels). This inotropic effect, however, was not accompanied by a lusitropic one since Ca^2+^ decay kinetics remained essentially unchanged following isoprenaline addition (Figure 3C, left and right panels). In general, an increase in depolarizing cytoplasmic Ca^2+^ could translate into prolonged field potential durations (FPDs) on MEAs. However, frequency-corrected FPDs in K_v_7.1-positive hiPSC-CMs were unaffected by short-term isoprenaline stimulation and the same result was obtained following forskolin or IBMX treatment (Figure 3D). In stark contrast, QTc_max_ intervals in K_v_7.1-deficient CMs—which were already increased compared to K_v_7.1-positive cells in unstressed conditions—were further elevated to high levels following short-term β-adrenergic stimulation or adrenoceptor-independent cAMP enhancement (Figure 3D). We conclude that *KCNQ1* expression not only restricts QT interval duration in the cardiomyocyte ground state but also balances QT prolongation under adrenergic stress conditions. Furthermore, these observations imply that K_v_7.1 becomes immediately activated upon adrenergic stimulation but again, it seemed enigmatic to us how this would be brought about given its low abundance at the plasma membrane.

### Adrenergic stress promotes fast K_v_7.1 recycling to the outer cardiomyocyte membrane

**Figure 4:**
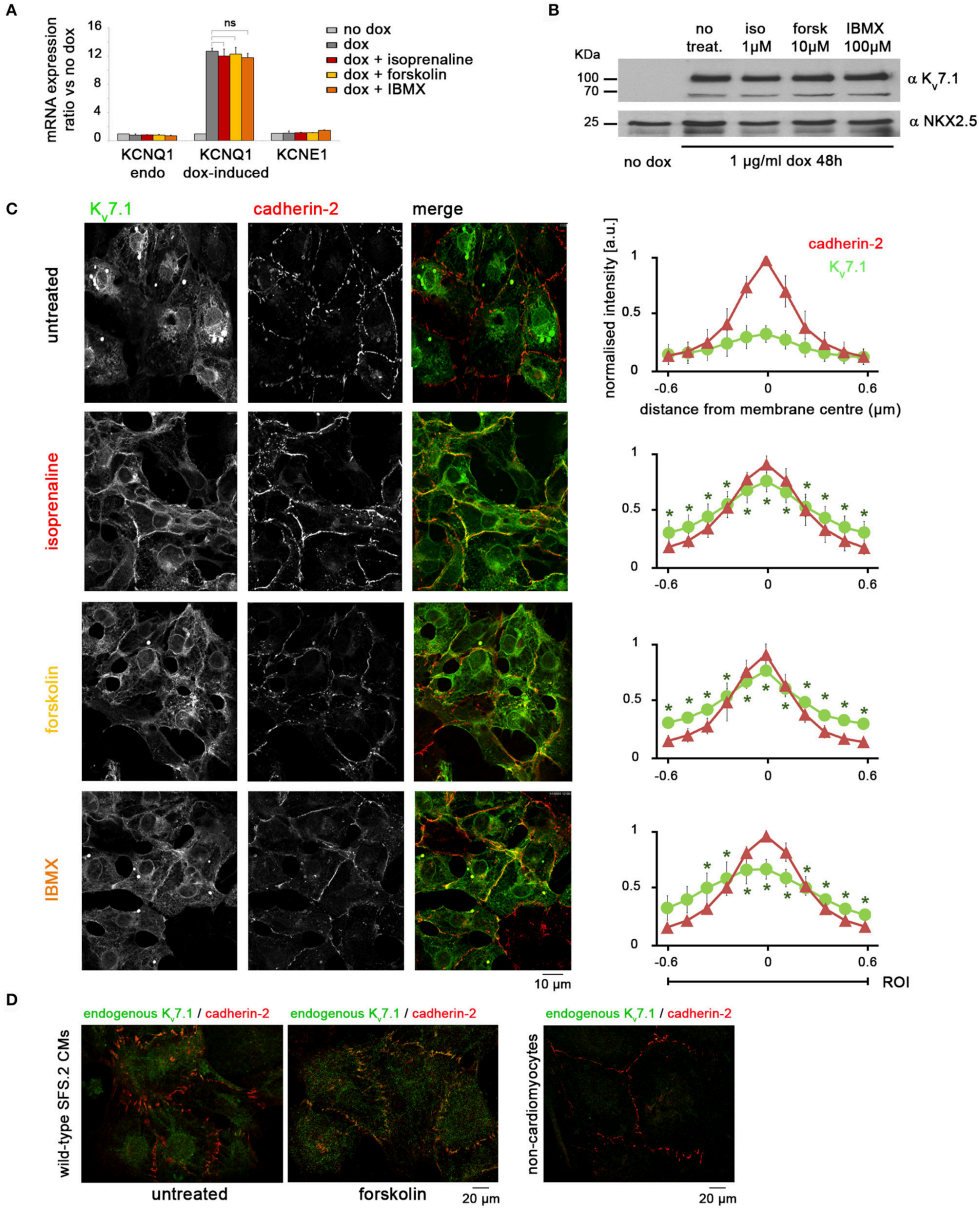
Adrenergic stimulation promotes rapid accumulation of K_v_7.1 at the outer cardiomyocyte membrane. **(A)** RT-qPCR analysis showing that treatments with isoprenaline (1 μM, 2′), forskolin (10 μM, 2′), and IBMX (100 μM, 10′) have no short-term effect on *KCNQ1* mRNA abundance (*n* = 3 biological replicates). **(B)** Immunoblot indicating that short-term stimulation with isoprenaline, forskolin, or IBMX does not affect total amount of K_v_7.1 protein. **(C)** Representative immunostainings (left) and quantitative analysis (right) showing that β-adrenergic agonists significantly promote a rapid (within 2 min) accumulation of dox-induce K_v_7.1 at the outer cardiomyocyte membrane (three independent experiments, eight images, 10–30 data points per image). Cadherin-2 served to identify cell boundaries. Pixel intensities were initially quantified at an absolute scale and are thus comparable between charts. ^*^ Indicates statistically significant differences compared to untreated cells. **(D)** Left: Immunostaining of endogenous K_v_7.1 in 5 week-old untreated and forskolin-treated wild-type CMs suggests a comparable stress-induced accumulation of the channel at the plasma membrane. The overall low K_v_7.1 abundance impedes accurate quantification. Right: As a staining control, a fraction of non-CMs present in the same cell preparations shows virtually no K_v_7.1 signal.

In exploring potential mechanisms, we considered short-term effects on *KCNQ1* mRNA levels, despite using a transgenic model. Short-term stimulation of dox-treated KCNQ1^fs/fs_K.TET−ON^-CMs with molecules mimicking β-adrenergic signaling, however, did neither increase (mutated) endogenous *KCNQ1* transcript levels nor exogenous ones (Figure 4A). Similarly, K_v_7.1 protein levels were unchanged after 2 min of activating cAMP signaling (Figure 4B). As a confirmation, cycloheximide treatments blocking K_v_7.1 translation suggested that the channel protein displayed low turnover rates within the first 20 min of exposure (data not shown). Alternatively to changes in total K_v_7.1 abundance, we then considered the possibility that cAMP enhancement may lead to a rapid translocation of K_v_7.1 from intracellular stores toward the outer cardiomyocyte membrane. Strikingly, isoprenaline, forskolin, and IBMX all promoted a significant accumulation of K_v_7.1 at the plasmalemma—within minutes or less—as evidenced by quantitative confocal immunofluorescence analysis (Figure 4C). Despite the technical difficulty in quantifying endogenous K_v_7.1 in maturated wild-type hiPSC-CMs, we still sought to confirm this striking result in a qualitative manner using native cells. Indeed, short-term (2 min) forskolin treatment of wild-type hiPSC-CMs following 5 weeks of maturation clearly caused an accumulation of endogenous K_v_7.1 at the plasma membrane, like in the dox-induced scenario (Figure 4D). These data reveal a novel mechanism of K_v_7.1 activation in human cardiac cells, namely, fast channel recycling as a short-term response to acute β-adrenergic stimulation.

### Fast K_v_7.1 recycling is RAB4A-dependent

**Figure 5:**
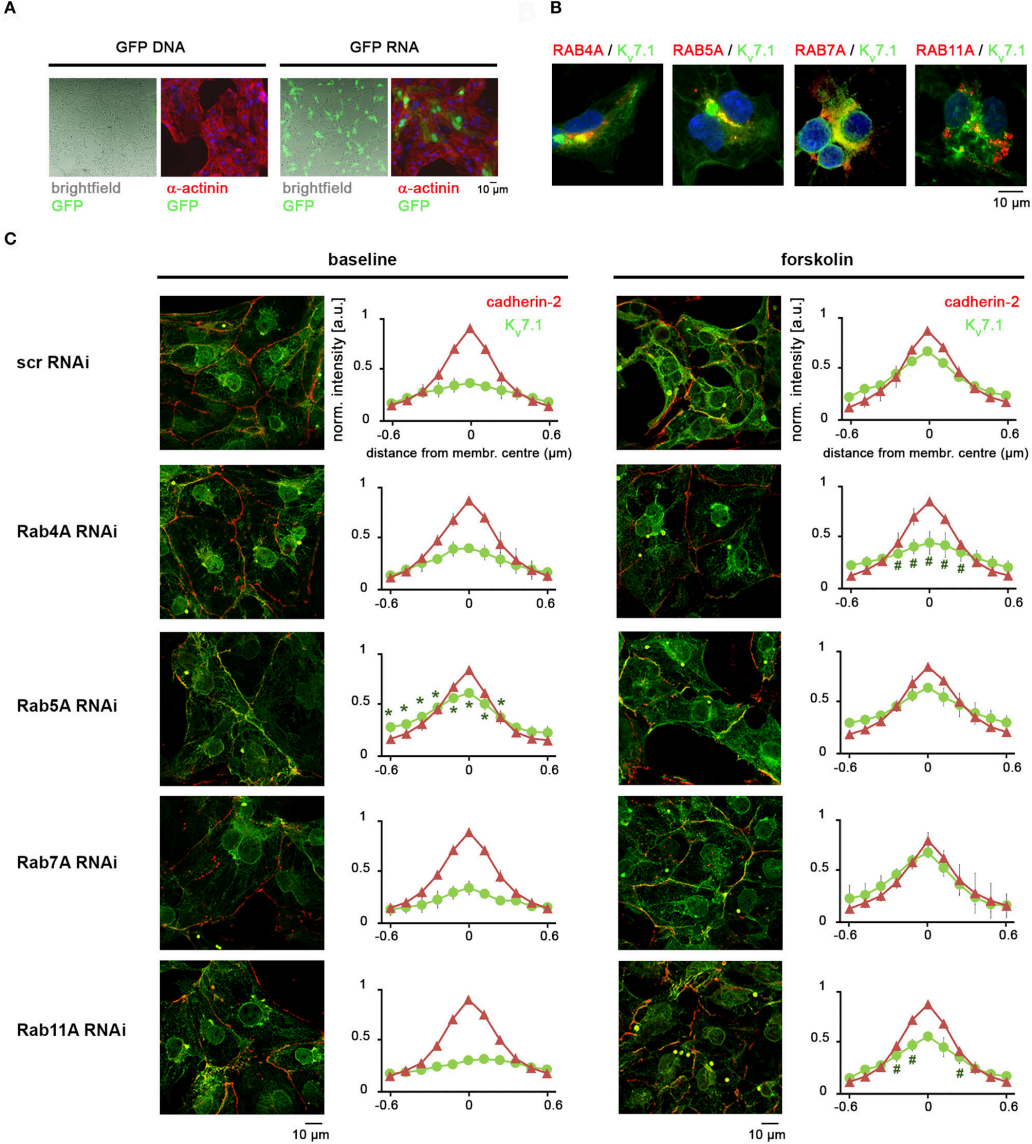
Fast stress-induced K_v_7.1 recycling is based on a RAB4A-dependent mechanism. **(A)** Pilot experiment showing that hiPSC-CMs may efficiently be transfected with polycistronic RNA molecules but essentially not with DNA plasmids. See methods section for RNA transfection conditions. A standard GFP-expressing plasmid was administered using routine conditions established for undifferentiated hiPSCs. **(B)** Overexpression of DsRed-RAB fusion RNA constructs in dox-treated KCNQ1^fs/fs_K.TET−ON^ CMs indicating partial co-localization with K_v_7.1 under baseline conditions. **(C)** RAB knockdown experiments under baseline and cAMP stress conditions (2 min forskolin). Left: Representative immunostainings. Right: Quantification of K_v_7.1 abundance at the outer cardiomyocyte membrane (two independent experiments, 4–8 images, 10–30 data points per image). ^*^Indicates significant differences in K_v_7.1 intensity compared to scrambled siRNA controls. Note in the RAB4A knockdown the decrease in K_v_7.1 plasma membrane abundancy after forskolin treatment, as compared to stressed control cells.

RAB GTPases are key mediators of intracellular trafficking and have previously been associated with K_v_7.1 recycling, albeit so far with mediating slow-rate ion channel turnover in non-cardiac cell contexts (Seebohm et al., 2007; Balse et al., 2012; Abbott, 2014; Andersen et al., 2015). hiPSC-CMs appeared to be refractory to the transfection of DNA constructs but we found that *in-vitro* transcribed mRNA could efficiently be delivered into these cells (Figure 5A). We hence prepared DsRed fusion transcripts of *RAB4A, RAB5A, RAB7A*, and *RAB11A* which are mediators of fast recycling, clathrin-based endocytosis, late endosome trafficking, and slow recycling, respectively (Balse et al., 2012). mRNA-based overexpression in KCNQ1^fs/fs_K.TET−ON^-CMs suggested that some of these factors were at least partially associated with K_v_7.1 (Figure 5B). Due to technical shortcomings perhaps owing to superphysiological expression levels of the transgenic RAB mRNAs, however, additional experiments under stress conditions remained rather inconclusive (not shown). Instead, we subsequently performed gene silencing of endogenous RABs using RNAi, to potentially reveal which of the aforementioned trafficking pathway(s) may be involved in the β-adrenergic response. mRNA knockdown efficiencies were above 70% in all cases (data not shown). Dox-treated KCNQ1^fs/fs_K.TET−ON^-CMs transfected with siRNAs against the four RABs or with scrambled controls were analyzed under baseline conditions as well as after short-term forskolin treatment, which revealed the following. RAB5A appears to mediate baseline K_v_7.1 endocytosis in hiPSC-CMs as its silencing leads to plasma membrane accumulation of the channel in unstressed conditions (Figure 5C, third panel, left). By contrast, RAB4A silencing did not affect K_v_7.1 abundance at the outer cardiomyocyte membrane in the CM ground state. Importantly, however, it strongly interfered with fast forskolin-driven K_v_7.1 recycling (Figure 5C, second panel, right). Knockdown of RAB11A exerted a similar but less pronounced effect, and RAB7A silencing did not at all affect K_v_7.1 trafficking (Figure 5C, last two panels). Collectively, these data reveal RAB4A as the key mediator of fast cAMP stress-induced K_v_7.1 recycling, while a minor additional contribution by RAB11A cannot be ruled out according to this analysis.

## Discussion

Several congenital diseases including LQT1 are associated with protein misfolding, result in impaired intrinsic protein functionality or lead to incorrect protein localization due to trafficking defects. Although misfolded ion channel proteins may generally be sorted out in a process called quality control and become degraded by the proteasome, in case of a genetic defect by far not all pathologically misfolded or mislocated proteins will be subject to this mechanism. This holds true for K_v_7.1 channels as well. Thus, disease pathology in LQT1 as a result of *KCNQ1* mutations can either be a consequence of a sheer protein loss as in many cases of JLNS or of compromised function at the appropriate subcellular compartment (Moretti et al., 2010; Zhang et al., 2014). There is emerging evidence that certain mutations in *KCNQ1* can indeed lead to altered endocytotic channel trafficking, with potentially devastating consequences for patients (Seebohm et al., 2008). For this reason as well as for understanding basic cardiac physiology, it is crucial to explore the functional relevance of K_v_7.1 and, particularly, the associated trafficking mechanisms in a human cardiomyocyte setting.

Specifically, the mechanistic understanding of β-adrenergic stress protection by K_v_7.1 in human cardiomyocytes has been incomplete until now. Here, we have used a *KCNQ1*-deficient JLNS model hiPS cell line that could optionally be complemented with the channel using small molecule-based transgene induction. This system hence enabled reliable K_v_7.1 localization and additionally allowed for comparing K_v_7.1-positive and deficient scenarios in otherwise identical cell populations. Indeed, *KCNQ1* induction readily promoted an expected QT-like interval reduction highlighting the functionality of the system and its relevance for LQTS/JLNS modeling.

**Figure 6:**
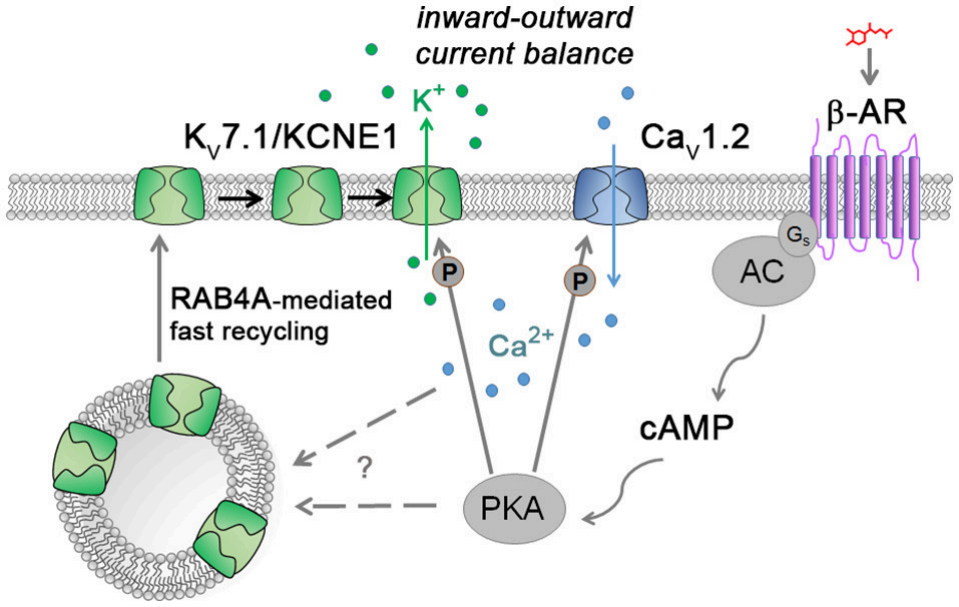
Model illustrating the suggested cAMP stress protection mechanism together with known ways of K_v_7.1/KCNE1 and Ca_v_1.2 modulation. Catecholamines stimulate, through the β-adrenergic receptor (β-AR), the production of the second messenger cAMP by adenylyl cyclase (AC) which in turn activates protein kinase A (PKA). Active PKA phosphorylates L-type calcium channels to enhance Ca^2+^ influx into cardiomyocytes. Simultaneously, K_v_7.1 channels are fast-recycled from the early vesicle reserve to the plasma membrane by means of the small GTPase RAB4A. Following channel accumulation at the outer cell membrane, K_v_7.1 becomes phosphorylated by PKA to further contribute to balancing the increased Ca^2+^ inward currents via K^+^ efflux. How exactly fast K_v_7.1 recycling is induced remains to be determined (“?”).

Moreover, sufficiently maturated hiPSC-CMs are responsive to adrenergic stimulation resulting in chronotropic as well as inotropic effects, here monitored as increased spontaneous contraction rates and calcium transient amplitudes, respectively. A third effect, lusitropy (faster relaxation and Ca^2+^ decline), was barely observed in our system perhaps reflecting an overall operative but still underdeveloped sarcoplasmic reticulum (SR) in hiPSC-CMs (Itzhaki et al., 2011). It is well-known that the PKA-mediated phosphorylation of the L-type voltage-gated calcium channel Ca_v_1.2 is key to promoting the inotropic effect (Figure 6) (Bers, 2002). The positive chronotropic response, by contrast, is less obvious as it is mediated through HCN channel modulation of pacemaker cells *in vivo* (Alig et al., 2009). hiPSC-CMs, however, tend to acquire a ventricular identity by default, yet they also show detectable funny current levels carried by HCN channels (Ma et al., 2011). This fact may on the one hand causally contribute to their spontaneous beating behavior but on the other, it may also help to explain why hiPSC-CMs actually show a chronotropic response to the sympathomimetic isoprenaline. Hence, although often considered a drawback, the specific features of hiPSC-CMs may also be exploited in a positive sense, for instance for investigating pleiotropic downstream effects of adrenergic stimulation.

*KCNQ1*-deficient hiPSC-CMs strikingly developed *TdP*-like arrhythmias upon adrenergic stimulation or more immediate cAMP enhancement, which closely resembles the patient scenario and confirms the overall rationale of β-blocker therapy in LQT1 (Schwartz et al., 2006). But how exactly are arrhythmias triggered under these conditions? Our data show that the cells are confronted with a three-fold challenge: Firstly, K_v_7.1- deficient CMs already display a pronounced FPD and action potential prolongation under baseline conditions, as also seen previously in patient-derived hiPSC-CMs (Zhang et al., 2014), implying that K_v_7.1 does also play a role in the CM ground state (Greber et al., 2015). Secondly, adrenergic stimulation induces a chronotropic effect that the cells have to cope with in terms of their repolarization kinetics. Finally, a third effect was surprising to us and can only be seen in a *KCNQ1*-deficient scenario as shown here: Isoprenaline or adrenoceptor-independent cAMP elevation promoted an additional FPD prolongation specifically in K_v_7.1-negative cells, which will further exacerbate the situation, to ultimately result in increased BVR or even *TdPs* (Thomsen et al., 2006; Oosterhoff et al., 2007). In their pioneering disease modeling work, Moretti et al. notably observed action potential prolongations following isoprenaline treatment specifically in LQT1 cells, which confirms our FPD data and extends its relevance from JLNS to LQT1 in general (Moretti et al., 2010). K_v_7.1/KCNE1 is believed to be a functional counterplayer of Ca_v_1.2 and both channels indeed become kinetically activated by PKA phosphorylation in wild-type CMs as part of the inotropic response (Figure 6) (Bers, 2002; Moretti et al., 2010). K_v_7.1 deficiency, however, will cause an imbalance of this otherwise well-coordinated system under adrenergic stress conditions, to result in Ca^2+^ overload from the SR and, consequently, BVR and *TdPs* (Thomsen et al., 2006; Johnson et al., 2013).

Conversely, *KCNQ1* induction fully prevented FPD prolongation and adverse effects on the macroscopic beating behavior of the cells under adrenergic stress conditions. In principle, the known PKA-based phosphorylation of membrane-bound K_v_7.1 could account for this protective effect. This mechanism alone, however, does not sufficiently explain the full extent of functional K_v_7.1/KCNE1 activation following β-adrenergic stimulation (Nicolas et al., 2008). Moreover, and first of all, a large fraction of ion channel protein would have to be localized to the correct subcellular compartment, i.e., the CM plasma membrane—which is simply not the case under baseline conditions. Notably, this conclusion based on the hiPSC-CM system is also supported by observations made in ventricular guinea pig CMs (Wang et al., 2013). Here, we reveal an additional mechanism of cAMP-mediated K_v_7.1 stimulation with particular relevance to acute stress scenarios. This is based on fast recycling in a mostly RAB4A-dependent manner. We propose that only by modulating channel trafficking this way can human cardiomyocytes cope with the profound challenges associated with the FFR - and likewise keep I_Ks_ conducted by K_v_7.1/KCNE1 at sufficiently low levels under resting conditions (Figure 6).

As a regulatory mechanism, it seems strategic that both channels, Ca_v_1.2 and K_v_7.1/KCNE1, are controlled by the same signaling pathway, the cAMP cascade, as this fact may best ensure a tight coupling of the two channel activities at all possible stress levels. Indeed, beat frequency-corrected FPDs were essentially invariant in stressed *KCNQ1*-expressing cells compared to unstressed ones, which highlights the robustness of this adaptation system. Finally, our data are not at odds with previously published K_v_7.1 trafficking mechanisms, notably RAB11-based slow recycling, as these may well play a role in ensuring homeostatic I_Ks_ levels in cardiomyocytes or other contexts (Seebohm et al., 2007; Andersen et al., 2015). Likewise, our results are not redundant with those by Wang et al. (2013) who proposed endoplasmic reticulum fragmentation as a slow-rate K_v_7.1 trafficking mechanism. In general, it is well conceivable that K_v_7.1 trafficking may be regulated at different levels in human cardiomyocytes, or in different ways across species.

In sum, our study reveals a key mechanism underlying acute adrenergic stress adaptation of human cardiomyocytes. Our findings are based on the hiPSC-CM system, thereby exemplifying its power for addressing fundamental questions of cardiac physiology. However, it is well-accepted that hiPSC-CMs are not identical to primary cardiomyocytes regarding their physiology and maturation status. It will be important, therefore, to potentially validate our hypotheses in adult human CMs. Moreover, our data also bear important implications for the pharmacological safety testing of drug candidates, as they suggest that acute stress conditions or a KCNQ1-deficient scenario should be routinely monitored in addition to the wild-type CM ground state (Colatsky et al., 2016; Huang et al., 2017). In future investigation, it will be interesting and essential to elucidate whether cAMP-induced fast K_v_7.1 recycling involves a novel and yet-to-be-identified PKA target or, alternatively, whether it is the PKA-mediated increase in cytoplasmic Ca^2+^ itself that triggers RAB4A-dependent exocytosis and, thereby, K_v_7.1 activity under acute adrenergic stress conditions (Figure 6) (Bartos et al., 2017). The former possibility may also imply novel means of manipulating the functional activity of K_v_7.1/KCNE1 in disease contexts.

## Author contributions

IP, BG, and GS designed experiments. IP and EF performed experiments. SF provided research material. IP, EF, FM, and BG analyzed the data. IP, BG, and GS wrote the manuscript with input from co-authors.

### Conflict of interest statement

The authors declare that the research was conducted in the absence of any commercial or financial relationships that could be construed as a potential conflict of interest.

## Supplementary material

Table S1 Oligonucleotides and DNA sequences used in this study.
